# Supplementary material for: A long-term epigenetic memory switch controls bacterial virulence bimodality
Source: eLife. 2017 Feb 7;6:e19599. doi: 10.7554/eLife.19599 (PMC5295817; doi:10.7554/eLife.19599)
Supplement: Supplementary file 1. — The relative copy number was obtained by coverage analysis of the whole genome sequencing data for BIG and SMALL bacteria (Goldberg et al., 2014). The WGS analysis reveals differences in EAF plasmid copy number only and the raw data is available as NCBI BioProject PRJNA255355 (Accessions: SRX757584 and SRX757585 for SMALL and BIG respectively). DOI: http://dx.doi.org/10.7554/eLife.19599.027 [file elife-19599-supp1.docx]

**Supplementary file 1. Whole genome sequencing of the BIG and SMALL morphotypes reveals differences in EAF plasmid copy number only.**

| **BIG** | | |
| --- | --- | --- |
| **Genomic compartment** | **Reference genome** | **Relative Copy**  **Number** |
| E2348/69 genome | 0127:H6 E2348/69 ( FM180568.1 ) | 1 |
| pMAR2 (EAF plasmid) | 0127:H6 E2348/69 plasmid pMAR2 (FM180569.1 ) | 0.37 |
| p5217 | E2348/69 plasmid p5217 | 6.71 |
| pE2348-2 | 0127:H6 E2348/69 plasmid pE2348-2 (FM180570.1) | 3.98 |
| **SMALL** | | |
| **Genomic compartment** | **Reference genome** | **Relative Copy**  **Number** |
| E2348/69 genome | 0127:H6 E2348/69 ( FM180568.1 ) | 1 |
| pMAR2 (EAF plasmid) | 0127:H6 E2348/69 plasmid pMAR2 ( FM180569.1 ) | 0.85 |
| p5217 | E2348/69 plasmid p5217 | 6.14 |
| pE2348-2 | 0127:H6 E2348/69 plasmid pE2348-2 (FM180570.1) | 4.1 |
