# Supplementary material for: A long-term epigenetic memory switch controls bacterial virulence bimodality
Source: eLife. 2017 Feb 7;6:e19599. doi: 10.7554/eLife.19599 (PMC5295817; doi:10.7554/eLife.19599)
Supplement: Supplementary file 2. — DOI: http://dx.doi.org/10.7554/eLife.19599.028 [file elife-19599-supp2.docx]

**Supplementary file 2. List of strains and plasmids used in this study.**

| **Strain or plasmid** | **Description** | **Reference or source** |
| --- | --- | --- |
| ***Cell lines*** | | |
| HeLa S3 | Human cervical adenocarcenoma line  [(RRID:CVCL_0058)](https://ncit.nci.nih.gov/ncitbrowser/ConceptReport.jsp?dictionary=NCI_Thesaurus&code=C4029) | ATCC (CCL-2.2) |
| ***EPEC strains*** | | |
| E2348/69 | EPEC wild type (strain O127:H6) | Levine et al., 1978 |
| JPN15/EAF^-^ | Derivative of E2348/69 cured of the EAF plasmid, pMAR2 | Gómez-Duarte and Kaper, 1995 |
| JPN15/EAF^+^ | JPN15 complemented with a transposon-marked EAF plasmid, pMAR7 | Girón et al., 2002 |
| EM371 *(Δler)* | E2348/69 *ler::kan* | Friedberg et al., 1999 |
| EM3715 *(ΔgrlRA)* | E2348/69 *grlRA::kan* | Padavannil et al., 2013 |
| *ΔperA* | E2348/69 *perA::kan* | This study |
| EM3716 *(ΔperC)* | E2348/69 *perC::kan* | This study |
| EM41 *(ΔbfpA)* | E2348/69 *bfpA::TnphoA* | Donnenberg et al., 1992 |
| EM2455 *ler-GFP* | E2348/69 containing a transcriptional fusion of PLEE1, *ler*, and *gfp* | Yerushalmi et al., 2008 |
| ***K-12*** | | |
| ME5000 | BW25113 derivative *F^-^ rpsL polA12 Zih::Tn10* | NBRP E. coli strain collection (Keio) |
| MG1655 | K-12 F^–^ λ^–^ *ilvG*^–^ *rfb-50* *rph-1* | CGSC (#6300) |
| ***Plasmids*** | | |
| pZS*1HGFP | pZ series (Lutz and Bujard, 1997) very low copy derivative, used for cloning | Rotem et al., 2010 |
| pZS11*GFP | pZS*1HGFP containing GFP expressed from P_LtetO-1_ promoter instead of *H* promoter | This study |
| pPerABC-GFP | pZS1H*GFP containing transcriptional fusion of *perABC* operon with its upstream regulatory region instead of *H* promoter | This study |
| pPerA-GFP | Derivative of pPerABC-GFP plasmid containing only *perA* gene with *perABC* upstream regulatory region | This study |
| pPerB-GFP | Derivative of pPerABC-GFP plasmid containing only *perB* gene with *perABC* upstream regulatory region | This study |
| pPerAB-GFP | Derivative of pPerABC-GFP plasmid containing both *perA* and *perB* gene with *perABC* upstream regulatory region | This study |
| pZA21mCherry | pZ series derivative plasmid containing mCherry expressed from P_LtetO-1_ promoter | Gefen et al., 2008 |
| pZA21YFP | pZ series derivative plasmid containing YFP expressed from P_LtetO-1_ promoter | This study |
| pSA11 | pBR322 derivative containing lacI^q^ and GFP expressed from *tac* promoter | Schlosser-Silverman et al., 2000 |
| pKD46 | Helper plasmid for λ Red recombineering | Datsenko and Wanner, 2000 |
| pMAR2 | Natural EAF plasmid of E238/69 | Baldini et al., 1983 |
| pMAR7 | Derivative of the native plasmid pMAR2 marked with Amp^r^ by transposition of Tn*801* | Baldini et al., 1983 |
