# Supplementary material for: A long-term epigenetic memory switch controls bacterial virulence bimodality
Source: eLife. 2017 Feb 7;6:e19599. doi: 10.7554/eLife.19599 (PMC5295817; doi:10.7554/eLife.19599)
Supplement: Supplementary file 3. — DOI: http://dx.doi.org/10.7554/eLife.19599.029 [file elife-19599-supp3.docx]

**Supplementary file 3. List of primers used in this study.**

| name | purpose | sequence |
| --- | --- | --- |
| PerA-KanR-intF | Reverse primer for *kan* insertion into *perA* region to produce perA::kan by λ Red recombination | AACACTCATTGGGACATGGAAATTGTCGGAATCGATAATAGTAATCCGTACAGAAAGAGTTGGTAGCTCAGAGAAC |
| PerA-KanF-intR | Forward primer for *kan* insertion into *perA* region to produce perA::kan by λ Red recombineering | TCTCAGACAAGAATCACTCATAAACATTGAACTACTGACATCGCCTAGTTTCCAAAGAGTTGGTAGCTCAGAGAAC |
| p_PABC_FN | Forward primer for *pPerABC* genomic region for isothermal cloning into pZS*1HGFP | TCGTCTTCACCTCGAGAGTTAAGTTATAACTGGGGC |
| p_PABC_RN | Reverse primer for *pPerABC* insert for isothermal cloning into pZS*1HGFP | CTCCTCTTTAATGAATTCCTGAGAGATCTACTATTCCG |
| pZS-isoFN | Forward primer for pZS*1HGFP vector for isothermal cloning of *pPerABC* | GAATTCATTAAAGAGGAGAAAGGT |
| GFP-PFWD | 5'-phosphorylated forward primer for excision of *perC* or *perBC* from pPerABC-GFP to produce pPerAB-GFP or pPerA-GFP, respectively | GTTCGGAATAGTAGATCTCTCA |
| PerB-endR | Revers primer for excision of *perC* from pPerABC-GFP, to produce pPerAB-GFP | TACTTGATAACCTATGGTGCTC |
| PPER_PREV | 5'-phosphorylated forward primer for excision of *perA* from pPerAB-GFP, to produce pPerB-GFP | ATTAACCACCTTCATGCGCGT |
| PerB-FWD | Forward primer for excision of *perA* from pPerAB-GFP, to produce pPerB-GFP | GAAGAACAATTTGAGAGAAGAGA |
| PerA-REV | Revers primer for excision of *perBC* from pPerABC-GFP, to produce pPerA-GFP | CTTATGGCAATGTTCCTTGTG |
| pZS-isoRN | Reverse primer for pZS*1HGFP vector for isothermal cloning of *pPerABC* | CTCGAGGTGAAGACGAAAGG |
| TetO-KpnI-FWD | P_LtetO-1_ promoter containing primer for cloning into pZS*1HGFP | TCCCTATACGTGATAGAGATTGACATCCCTATCAGTGATAGAGATACTGAGCACATCAGCAGGACGCACTGACCGAATTCATTAAAGAGGAGAAAGG |
| pZPhos-REV | 5'-phosphorylated primer for P_LtetO-1_ promoter cloning into pZS*1HGFP | CTCGAGGTGAAGACGAAAGG |
